# Perceptions and barriers to dietary fiber intake among middle-aged south Indian women: A qualitative study

**DOI:** 10.1371/journal.pgph.0006685

**Published:** 2026-06-24

**Authors:** Suvarna Hebbar, Shashikiran Umakanth, Latha Thimmappa, Amrutha M. S., Ramya Bhagavath

**Affiliations:** 1 Clinical Nutrition and Dietetics Program, Manipal College of Health Professions, Manipal Academy of Higher Education, Manipal, Karnataka, India; 2 Department of General Medicine, Dr. TMA Pai Hospital, Udupi, Melaka Manipal Medical College, Manipal Academy of Higher Education, Manipal, Karnataka, India; 3 College of Nursing, All India Institute of Medical Sciences (AIIMS), Bibinagar, Telangana, India; 4 Department of Clinical Nutrition and Dietetics, Kasturba Hospital, Manipal, Karnataka, India; Universitat Rovira i Virgili, SPAIN

## Abstract

Dietary fiber plays a critical role in preventing non-communicable diseases (NCDs), yet intake remains low among many Asian women. In India, the average dietary fiber intake is approximately 15–20 g/day, substantially below the recommended range of 25–30 g/day, and fewer than 10–15% of adults meet the recommended daily fiber intake. Rapid urbanization and changing food environments, including increased availability of ultra-processed foods and affordability challenges, may reduce consumption of fiber-rich foods such as whole grains, fruits, and vegetables.In South Asian households, women play a central role in food purchasing and preparation, but time constraints, economic, and household responsibilities may influence dietary behaviors and fiber intake. This qualitative study aimed to explore the perceptions, barriers, and enabling factors influencing dietary fiber consumption among middle-aged South Indian women, specifically comparing those with high versus low fiber intake patterns.An exploratory qualitative design was employed, utilizing in-depth, semi-structured interviews with 13 purposively sampled women (aged 35–55) from a tertiary care hospital in South India. Participants were categorized into high-fiber (n = 7, ≥ 20 g/day) and low-fiber (n = 6, < 20 g/day) groups based on 24-hour dietary recall. Data, collected in Kannada and transcribed verbatim, were analyzed thematically using NVivo 15, adhering to COREQ guidelines.Six major themes emerged: cultural/routine-based preferences, dietary practices/behaviours, economic influences, knowledge-related factors, physical activity/lifestyle constraints, and psychosocial determinants. High-fiber consumers exhibited greater dietary awareness, structured meal planning, adherence to traditional practices, and stronger family support. In contrast, low-fiber consumers faced time constraints, limited nutritional knowledge, reliance on convenience foods, and greater psychological strain, often compromising dietary quality.Dietary fiber intake among South Indian women is shaped by complex interactions of cultural norms, socioeconomic status, lifestyle, and psychosocial factors, transcending mere nutritional knowledge. Interventions must be culturally sensitive, gender-responsive, and address systemic barriers, empowering women to improve family nutrition and combat NCDs effectively.

## Introduction

Dietary fiber, a non-digestible component of plant-based foods, plays a critical role in human health by improving gastrointestinal function and regulating glucose and lipid metabolism, thereby helping prevent and manage non-communicable diseases such as type 2 diabetes, cardiovascular diseases, obesity, and certain cancers [1,2]. Many low- and middle-income countries, including India, are experiencing the triple burden of malnutrition, characterized by the coexistence of undernutrition, micronutrient deficiencies, and overweight/obesity, posing significant public health challenges [3]. This shift is driven by changing dietary patterns, including reduced consumption of fiber-rich traditional foods and increased preference for energy-dense ultra-processed foods, contributing to low dietary fiber intake [4].Urbanization and changing lifestyles have further altered traditional dietary practices, increasing reliance on convenience foods and reducing intake of traditional high-fiber foods. The affordability and accessibility of healthier food options remain important barriers for many households, influencing dietary choices and the consumption of fiber-rich foods such as fruits, vegetables, legumes, and whole grains.

Women, particularly in Asian households, play a key role as primary decision-makers in food choices, influencing food purchasing, meal planning, and family dietary habits [5,6]. Although dietary challenges affect both men and women, women were specifically selected for this study due to their central role in household dietary decision-making and their higher vulnerability to metabolic disorders associated with dietary patterns [7]. However, unexpectedly, women often experience disproportionately high rates of nutrition-related health issues, including elevated incidences of overweight and obesity across both urban and rural Asian populations [5,8]. Middle-aged women also experience important life-stage transitions, including hormonal changes, pregnancy history, menopause, and psychosocial stress, which may influence dietary behaviors and nutritional intake, including fiber consumption [9]

Despite the well-established health benefits and recommended daily intake of 25–30 grams for adults by the World Health Organization and national guidelines, average fiber consumption among many Asian populations remains persistently below recommended levels [10,11]. This persistent gap is complicated by cultural norms, aggressive food marketing, limited availability and affordability of high-fiber foods, and widespread misconceptions [12]. Many consumers perceive fiber-rich foods as lacking in flavor, inconvenient, or incompatible with contemporary lifestyles [13]. Although previous studies in India have examined general dietary patterns and nutrient intake, limited research has explored the contextual factors influencing dietary fiber consumption among middle-aged South Indian women. In particular, little is known about how perceptions, household influences, cultural practices, and practical constraints differ between women with high and low dietary fiber intake. Exploring these contrasting experiences is important for understanding the underlying decision-making processes that shape fiber consumption and for informing culturally appropriate strategies to improve fiber intake among women [14]. The specific relationship of these complex factors and the decision-making processes influencing fiber consumption among South Indian women, particularly distinguishing between high and low intake patterns, remains underexplored. Hence, this study used a qualitative approach to explore the factors and decision-making processes influencing fiber consumption among women with high versus low intake patterns among middle-aged South Indian women [15].

## Methodology

This study employed an exploratory qualitative design using in-depth interviews to examine the dietary behavior and decision-making processes related to fiber intake among middle-aged South Indian women. The methods and findings of the study were reported using COREQ (Consolidated Criteria for Reporting Qualitative Studies) guidelines [16].

### Ethical considerations

Institutional ethics committee approved the study protocol. Written informed consent was obtained from all participants prior to their involvement in the study, and confidentiality was maintained throughout the research process. The study was conducted in accordance with ethical research principles.

### Study design, setting, and participants

Participants were purposively sampled from women aged 35–55 years who attended the outpatient departments of Medicine, Gynaecology, and Endocrinology at a tertiary care hospital in South India. Dietary fiber intake was estimated using a 24-hour dietary recall. Based on the estimated intake, participants were broadly categorized into two groups: those consuming ≥20 g/day of dietary fiber were classified as the relatively higher fiber intake group (n = 7), while those consuming <20 g/day were classified as the relatively lower fiber intake group (n = 6). Purposive sampling was used to recruit participants with relatively higher and lower dietary fiber intake based on dietary recall assessment. This grouping served as a qualitative sampling strategy to capture diverse perspectives and explore differences in perceptions, barriers, and dietary behaviors related to fiber consumption during the in-depth interviews, and was not intended for statistical comparison.

**Fig 1:**
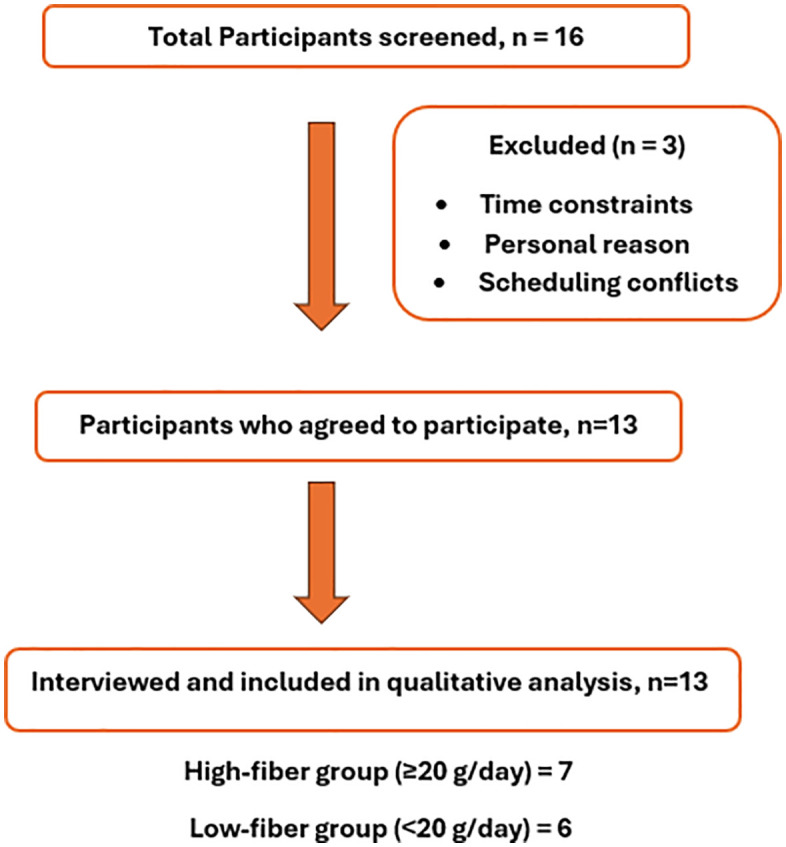
Participant flow diagram illustrating recruitment, screening, and inclusion of participants in qualitative study.

Participants were categorized based on dietary fiber intake using a pragmatic threshold of ≥20 g/day to define relatively higher fiber intake. Although the recommended intake for adults is 25–30 g/day, the cut-off of 20 g/day was selected based on findings from the prevalence study (N = 440), where the mean intake was approximately 20 g/day (median: 19.2 g/day), reflecting the typical consumption level in this population. This threshold enabled the identification of comparatively higher fiber consumers for the qualitative phase of the study.The primary focus of the study remained a qualitative exploration of participants’ perceptions, barriers, and dietary practices related to fiber consumption. A total of 13 participants were included in the study. This qualitative study was conducted as part of a broader research project investigating dietary fiber intake and metabolic health among overweight and obese women.The participant recruitment and inclusion process is illustrated in **Fig 1**.

### Data collection procedures & instruments

A semi-structured interview guide was developed based on a comprehensive literature review and expert consultation. The guide included 12 open-ended questions with follow-up probes covering dietary habits, knowledge of dietary fiber, cultural influences, food preferences, perceived barriers to fiber intake, and potential facilitators. The interview guide was reviewed and validated by six subject experts, and their feedback was incorporated to improve clarity, relevance, and cultural appropriateness. The semi-structured interview guide used in the study is provided as Supplementary Material (S1).

Interviews were conducted in Kannada, the native language of the participants, to facilitate comfortable communication and richer expression of experiences. Each interview lasted approximately 30 minutes and was conducted in a quiet and private space within the hospital premises to ensure participant comfort and confidentiality, even though participants were recruited from hospital outpatient departments. Efforts were made to maintain a relaxed and non-clinical interview environment.

All interviews were audio-recorded with participants’ consent. The interviews were conducted by trained researchers with backgrounds in public health and qualitative research methods. The interviewers had no prior personal relationship with the participants, which helped minimize potential bias during the data collection process.

### Data transcription and translation

All audio recordings were transcribed verbatim and subsequently translated into English by bilingual researchers with a healthcare background. Translations were cross-checked by another bilingual researcher to preserve semantic accuracy and cultural nuance.

To enhance credibility, transcripts and emerging interpretations were reviewed by the research team to ensure that the themes accurately reflected participants’ perspectives.

### Data analysis

All audio-recorded interviews were transcribed verbatim and translated into English for analysis. Data analysis was conducted using an inductive thematic analysis approach and facilitated by NVivo Version 15 software. Initially, all transcripts were read repeatedly to ensure familiarity with the data and to gain an overall understanding of participants’ perspectives.

An open coding process was then undertaken, during which meaningful segments of text were identified and assigned preliminary codes reflecting participants’ perceptions, experiences, and behaviors related to dietary fiber intake. Similar codes were subsequently grouped into broader categories through an iterative comparison process. These categories were further examined to identify patterns and relationships, leading to the development of overarching themes representing the key determinants of dietary fiber consumption.

NVivo software was used to organize transcripts, manage coding nodes, retrieve coded segments, and support systematic comparison between the high-fiber and low-fiber groups. Coding matrices and query tools within NVivo facilitated pattern identification and thematic organization.

To ensure analytical reliability, two researchers independently coded the interview transcripts and developed preliminary codes. Discrepancies in coding were discussed and resolved through consensus. Where necessary, a third senior researcher reviewed the coding to help resolve disagreements and ensure consistency in the thematic analysis.

Data analysis was conducted using a deductive coding approach guided by the study objectives and interview guide. Data saturation was assessed during the coding process and was considered achieved when no new codes or themes emerged from successive interviews. In this study, saturation was reached by the twelfth interview, and one additional interview confirmed that no new themes were identified.

The analysis focused on identifying commonalities and differences in perceptions, barriers, and enablers between the two fiber intake groups. While fiber intake values were used to categorize participants into comparison groups, the analysis itself focused on qualitative thematic interpretation of participants’ experiences, perceptions, and behaviors rather than quantitative comparisons.

## Results

### Participant characteristics

Table 1 summarizes the demographic characteristics of the 13 participants included in the study. Participants were aged 35–55 years and comprised seven women in the high-fiber intake group and six women in the low-fiber intake group. Characteristics including age, education level, occupation, household structure, and BMI status are presented to provide contextual background for the interpretation of the qualitative findings.

**Table 1:** Participant characteristics of study participants (n = 13).

Characteristic	High Fiber Group (n = 7)	Low Fiber Group (n = 6)
**Age**		
Mean age (years)	44.3	38.5
Age range (years)	35–54	36–43
**Fiber intake**		
Mean fiber intake (g/day)	25.2	15.5
**Family profile**		
Mean family size	5.1	5.5
**Household type**		
Nuclear/semi-nuclear	4 (57.1%)	2 (33.3%)
Joint family	3 (42.9%)	4 (66.7%)
**Occupation**		
Housekeeping staff	1 (14.3%)	1 (16.7%)
General store worker	2 (28.6%)	–
Staff nurse / Ward sister	2 (28.6%)	2 (33.3%)
Clerical/Associate	1 (14.3%)	3 (50.0%)
Psychologist	1 (14.3%)	–
**Education level**		
Master’s degree	1 (14.3%)	–
Graduate degree	6 (85.7%)	3 (50.0%)
Below graduate	–	3 (50.0%)
**BMI status**		
Overweight	1 (14.3%)	1 (16.7%)
Obese	6 (85.7%)	5 (83.3%)

Beyond the demographic characteristics presented in Table 1, qualitative findings revealed differences in household dynamics and dietary awareness between the two groups. Participants in the high-fiber group frequently reported greater autonomy in meal planning and stronger family support for preparing healthier meals. In contrast, participants in the low-fiber group often described limited control over household food decisions and competing family preferences that influenced their dietary choices.Variables such as BMI were included to provide general participant context and were not used as analytical variables in the qualitative thematic analysis (Table 2).

**Table 2:** Summary of key themes and subthemes identified from thematic analysis of participant interview.

Theme	Subtheme
Cultural and Routine-Based Food Preferences	Traditional Dietary Practices
Dietary Practices and Behaviours	a. Balanced and Thoughtful Cooking Practicesb. Seasonal Fruit Consumptionc. Selective Use of Millets Based on Experienced. Mindful and Health-Conscious Eating Practicese. Non-Vegetarian Dietary Practicesf. Planned and Balanced Eating Behaviourg. Reduced Dietary Diversity
Economic Influences on Dietary Choices	a. Food Waste Avoidanceb. Groceries and Need-Based Purchasing
Knowledge-Related Barriers and Enablers	a. Knowledge About Fiberb. Digital Media as a Source of Knowledgec. Beliefs About Genetic Healthd. Dietary Modification Based on Beliefs
Physical Activity and Lifestyle Constraints	a. Physical Activityb. Time-Related Lifestyle Barriers to Healthy Eating
Psychosocial Determinants of Dietary Behavior	a. Household Responsibilities and b. Emotional Drivers of Eating Behaviorc. Family-Influenced Dietary Decisions

### Theme 1. Cultural and routine-based food preferences

#### Traditional dietary practices.

High fiber participants showed stronger adherence to traditional vegetarian diets, structured meal routines, and culturally rooted food practices, which appeared to support regular intake of fiber-rich foods. Religious customs, fasting practices, and traditional household food patterns contributed to healthier eating behaviors.

*“We mostly eat vegetarian food and rarely consume non-vegetarian dishes. We have non-veg only on Sundays...” WH1 (High Fiber)*

Some participants described festival-related dietary restrictions that reinforced traditional eating practices.

*“During festivals like Ganesh Chaturthi... we don't use onion and garlic during that time.” WH5 (High Fiber)*

Fasting routines among high fiber participants often included fruits and simple home-prepared meals, reflecting greater dietary discipline and continuity of traditional practices.

*“I do fast on Fridays and Saturdays... proper meal in the afternoon, and at night, fruits.” WH4 (High Fiber)*

Traditional millet-based foods such as ragi were commonly consumed within families, including by children, indicating sustained use of culturally familiar high-fiber foods.

*“We mainly consume ragi... even kids drink ragi before school.” WH1 (High Fiber)*

Some participants also modified traditional recipes to improve health quality while preserving cultural preferences.

*“We don't use much sugar... I add jaggery instead.” WH1 (High Fiber)*

In contrast, low fiber participants reported less consistent adherence to traditional dietary practices and greater reliance on convenience foods and outside eating. Cultural food practices were often followed occasionally or only by older family members.

*“Only on Fridays, my mother-in-law follows vegetarian food...” WL2 (Low Fiber)*

Many participants associated modern lifestyles with reduced dependence on traditional meals and increased consumption of readily available foods, which appeared to lower fiber intake.

*“Nowadays, because of busy schedules, we mostly prepare whatever is easy and quick.” WL3 (Low Fiber)*

This highlights how changing lifestyle patterns and time constraints have reduced adherence to traditional home-prepared food practices, contributing to lower intake of fiber-rich foods.

*“All of us have already adjusted to today's lifestyle. There's nothing like strictly traditional food anymore.” WL4 (Low Fiber)*

Work schedules and irregular routines also contributed to inconsistent fasting and unplanned eating habits.

*“When I go to work, I don't usually fast much... I eat whatever I get.” WL1 (Low Fiber)*

Overall, the findings suggest that retention of traditional dietary routines and culturally embedded food practices may positively influence dietary fiber intake among middle-aged women.

### Theme 2. Dietary practices and behaviours

#### Balanced and thoughtful cooking practice.

High fiber participants reported using healthier cooking methods such as boiling and steaming, reflecting more conscious and balanced food preparation practices.

*“We mostly use boiling as our primary cooking method... occasionally steam food... like pundi.” WH1 (High Fiber)*

In contrast, low fiber participants described food choices driven mainly by convenience and availability, with less focus on nutritional quality.

*“Whatever is easily available, like fruits or vegetables, we just get that and cook.” WL2 (Low Fiber)*

#### Seasonal fruit consumption.

Seasonal fruit consumption was reported in both groups; however, high fiber participants demonstrated more consistent daily incorporation of fruits into their regular dietary routines.

*“Sweet lime and oranges are on daily basis...” WH7 (High Fiber)*

#### Selective use of millets based on experience.

Millet consumption in both groups was influenced by personal experiences, perceived health benefits, and family preferences, resulting in selective rather than routine use. High fiber participants associated millet intake with health promotion, whereas low fiber participants reported limited use due to household taste preferences and acceptability.

*“My sister gave me a millet powder for immunity... I mix 1.5 spoons with milk every day.” WH7 (High Fiber)*

*“I make things using wheat and jowar; ragi is used less... kids don't like it.” WL4 (Low Fiber)*

#### Mindful and health-conscious eating practices.

High fiber participants described mindful and health-oriented dietary practices, including the use of traditional herbal beverages as part of routine health maintenance. In contrast, low fiber participants reported habitual tea consumption primarily for stress relief and symptom management, reflecting emotionally driven dietary behaviors.

*“After washing his hands, my husband drinks jeera kashaya.” WH1 (High Fiber)*

*“I drink a lot of tea; if I don’t drink tea, I get a slight headache. I tend to have a bit more tea like that. Drinking more tea helps me relax a little.” WL6 (Low Fiber)*

#### Non-vegetarian dietary practices.

Low fiber participants reported frequent consumption of non-vegetarian foods with limited emphasis on accompanying vegetable intake, reflecting lower dietary diversity. In contrast, high fiber participants described a more balanced approach, emphasizing the inclusion of leafy greens and vegetables alongside non-vegetarian foods and moderation in dietary choices.

*“We eat non-veg 5–6 days a week.” WL2 (Low Fiber)*

*“It is better to include more leafy greens and vegetable in diet... I believe that eating egg in moderation is fine.” WH2 (High Fiber)*

#### Planned and balanced eating behaviour.

Participants in the high fiber group demonstrated proactive meal planning and structured food preparation practices that supported regular intake of fiber-rich traditional foods. Preparing ingredients in advance and following routine-based cooking practices appeared to facilitate healthier dietary consistency:

*“I soak rice and urad dal together... keep it overnight.” WH6 (High Fiber)*

In contrast, participants in the low fiber group reported irregular meal patterns influenced by time constraints, resulting in skipped meals and greater reliance on convenient snack-based foods. Limited dietary planning and reduced meal variety reflected the impact of busy lifestyles on maintaining balanced eatingbehaviors

*“I eat breakfast only when I have time; otherwise, I go for a snack or just stick to three main meals.” WL4 (Low Fiber)*

#### Reduced dietary diversity.

Participants in the low fiber group reported limited dietary diversity, with frequent reliance on repetitive and monotonous meal patterns. The preference for simple, routinely prepared foods reflected reduced variation in food choices, which may contribute to lower intake of fiber-rich foods and overall nutritional imbalance. Such practices also appeared to be influenced by convenience and habitual cooking patterns

*“We usually don't make too many varieties, mostly we prepare simple meals like rice ganji. Once we make ganji we eat for a couple of days.” WL3 (Low Fiber)*

### Theme 3. Economic influences on dietary choices

#### Food waste avoidance.

Participants from both fiber intake groups emphasized minimizing food waste, although their approaches differed.

Low-fiber participants described consuming home-grown vegetables mainly to avoid wastage, reflecting an economically driven and practical approach to food use rather than a focus on fiber quality or dietary diversity. One participant stated:

*“We grow vegetables at home... we avoid waste and eat them.” WL4 (Low Fiber)*

In contrast, high-fiber participants reported more planned purchasing behaviors, such as buying vegetables according to daily needs to reduce spoilage and ensure freshness. This reflected greater dietary planning and regular inclusion of fresh foods in meals. As one participant explained:

*“We buy only what we need for the day so that nothing goes to waste and we eat fresh vegetables daily.” WH3 (High Fiber)*

Some participants also highlighted purchasing locally available produce in small quantities, suggesting that affordability and convenience influenced food choices. One participant noted:

*“We don't go to check the quality, everything is brought loose like half kg at a time.” WH3 (Low Fiber)*

These findings indicate that economic considerations, including waste reduction, affordability, and purchasing practices, played an important role in shaping dietary choices across participants.

#### Groceries & need-based purchasing.

Participants described grocery purchasing as a balance between nutritional value, affordability, household needs, and family preferences.

High-fiber participants demonstrated more conscious purchasing behaviors, prioritizing foods that were both nutritious and economically beneficial. Their decisions reflected an effort to optimize dietary quality while remaining cost-conscious. One participant stated:

*“Whatever is cost-effective and nutritious, we purchase more of it.” WH1 (High Fiber)*

Low-fiber participants emphasized purchasing groceries in limited quantities based on immediate household requirements. This approach was associated with maintaining food quality, ease of storage, and avoiding unnecessary expenditure or waste. As one participant explained:

*“We make sure the groceries are of good quality and enough for use, but we don't buy in large quantities because it's easier to store when we buy only what's needed.” WL2 (Low Fiber)*

Family food preferences also emerged as an important determinant of women’s dietary practices. Participants reported that meal preparation was frequently modified according to the tastes of spouses and children, which at times restricted the regular inclusion of fiber-rich foods such as whole grains, vegetables, and legumes. Preference for refined grains and less fibrous foods among family members created challenges in maintaining high-fiber dietary patterns within the household.

### Theme 4. Knowledge-related barriers and enablers

#### Beliefs about genetic health.

Beliefs regarding hereditary risk influenced participants’ perceptions of obesity and dietary behaviors differently across fiber intake groups.

High-fiber participants perceived family history of obesity as a motivating factor to adopt healthier eating practices. Awareness of genetic susceptibility appeared to encourage greater dietary responsibility and intentional inclusion of nutrient- and fiber-rich foods. One participant stated:

*“I feel my obesity might be hereditary since my mother and sibling are also overweight, so I make a conscious effort to eat healthy by including more vegetables, pulses, fruits, and other nutritious foods in my diet.” WH2 (High Fiber)*

In contrast, low-fiber participants often attributed weight gains predominantly to hereditary factors, reflecting a more passive perception of obesity risk. This belief appeared to reduce emphasis on modifiable lifestyle practices, including adherence to a healthy, fiber-rich diet. As one participant explained:

*“Speaking abo\ut obesity, I have been gaining weight recently. Previously as well, and my father is obese, so it could be hereditary.” WL6 (Low Fiber)*

These findings suggest that while awareness of genetic predisposition can function as a facilitator for healthier dietary behaviors among high-fiber participants, it may also contribute to a sense of inevitability and reduced dietary motivation among low-fiber participants.

#### Dietary modification based on beliefs.

Low-fiber participants demonstrated a basic but limited understanding of dietary fiber, primarily associating it with fruits, vegetables, and leafy greens. Their responses reflected partial nutritional awareness, with fiber knowledge confined to commonly recognized food sources rather than a broader understanding of diverse fiber-rich foods and their health benefits. One participant stated

*“According to me, fiber is in fruits and vegetables... also leafy greens.” WL3 (Low Fiber)*

This suggests that incomplete knowledge regarding dietary fiber may influence food choices and limit the consistent incorporation of a wider variety of fiber-rich foods into daily diets.

#### Digital media as a source of knowledge.

High-fiber participants identified digital and social media platforms as important sources of nutrition-related information and health guidance. Their narratives indicated a proactive approach to seeking dietary knowledge, with online resources being used to enhance awareness and support healthier lifestyle practices. One participant explained:

*“With health being important, we often turn to social media for tips and advice. Since staying healthy is important, we try to learn more about it.” WH1 (High Fiber)*

This finding suggests that digital media served as an accessible avenue for acquiring health information and reinforcing positive dietary behaviors among high-fiber participants.

#### Knowledge about fiber.

Participants demonstrated varying levels of awareness regarding dietary fiber and its role in healthy eating.

Among low-fiber participants, knowledge of fiber appeared limited and was often reflected in dietary patterns characterized by a predominance of refined rice-based meals and frequent fried non-vegetarian preparations, with comparatively low inclusion of fiber-rich foods. Although some participants recognized pulses and legumes as sources of fiber, their understanding of fiber-rich foods remained restricted. One participant stated:

*“We should use more pulses and legumes because they have more protein and fiber, beyond that, I don't know much.” WL5 (Low Fiber)*

In contrast, high-fiber participants exhibited greater familiarity with fiber-containing foods and their perceived benefits. Fiber was commonly associated with traditional whole grains and satiety-promoting properties. As one participant explained:

*“Fiber is now ragi... gives us a full feeling...” WH5 (High Fiber)*

These findings indicate that broader awareness of fiber sources and their functional benefits may facilitate healthier dietary choices, whereas limited knowledge may contribute to lower consumption of fiber-rich foods.

### Theme 5. Physical activity and lifestyle constraints

#### Physical activity.

Marked differences in physical activity practices were observed between the fiber intake groups.

Low-fiber participants frequently reported sedentary lifestyles, attributing limited physical activity to work demands, fatigue, and lack of available time. These competing responsibilities often reduced opportunities for regular exercise and engagement in health-promoting behaviors. One participant stated:

*“There's no extra physical activity... no time or energy left.” WL3 (Low Fiber)*

In contrast, high-fiber participants described incorporating structured physical activity into their daily routines. Activities such as yoga and walking were consciously integrated into their schedules, reflecting a proactive approach toward maintaining health and overall well-being. As one participant explained:

*“I wake up at 4:30 and do 15 minutes of yoga, then go for a walk.” WH5 (High Fiber)*

These findings suggest that regular engagement in physical activity was more commonly observed among high-fiber participants, whereas lifestyle constraints and perceived fatigue acted as barriers to maintaining an active lifestyle among low-fiber participants.

#### Time-related lifestyle barriers to healthy eating.

Time availability emerged as an important factor influencing dietary practices, particularly among low-fiber participants. Balancing occupational responsibilities with household duties often left limited time for meal planning, food preparation, and personal health management. One participant described the competing demands on her daily routine:

*“Most time goes in the office... then at home with chores. Hard to get time for myself.” WL4 (Low Fiber)*

Busy schedules also contributed to irregular eating patterns, with participants reporting a lack of fixed meal timings. Such inconsistencies may hinder the regular consumption of balanced meals and reduce opportunities to incorporate fiber-rich foods into the daily diet. As one participant explained:

*“We just eat when the time comes... sometimes no fixed mealtime.” WL1 (Low Fiber)*

In contrast, high-fiber participants demonstrated greater use of anticipatory planning strategies to maintain healthy eating practices despite time constraints. Advance preparation of ingredients was commonly employed to facilitate the inclusion of nutritious foods within demanding schedules. One participant stated:

*“Since we leave early, I chop vegetables the night before.” WH5 (High Fiber)*

These findings suggest that while time pressures acted as a barrier to healthy eating among low-fiber participants, effective planning and meal preparation practices served as important enablers of healthier dietary behaviors among high-fiber participants.

### Theme 6. Psychosocial determinants of dietary behaviour

#### Household responsibilities and emotional drivers of eating behaviour.

Household dynamics and caregiving responsibilities played a significant role in shaping dietary behaviors and food-related decision-making.

Among high-fiber participants, shared household responsibilities were perceived as facilitators of healthier eating practices by creating time for meal planning and food preparation. One participant noted:

*“My mother-in-law and I divide work, so I get some time to plan and cook properly.” WH1 (High Fiber)*

At the same time, the absence of support systems was associated with increased workload and emotional strain, making dietary management more challenging. As one participant explained:

*“I must manage everything by myself. It becomes hectic.” WH1 (High Fiber)*

Parental concerns also influenced food choices. High-fiber participants reported intentionally limiting outside food consumption to maintain diet quality and prevent the development of unhealthy eating habits among children. One participant stated:

*“We rarely eat outside. We avoid it because the children might develop a habit.” WH4 (High Fiber)*

In contrast, low-fiber participants described a more responsive approach to outside food consumption, often influenced by children’s requests. As one participant noted:

*“Only if kids ask, we eat from outside.” WL2 (Low Fiber)*

Similarly, the introduction of fruits was described in modest and occasional terms, suggesting a lower prioritization of fiber-rich foods within routine dietary practices. One participant reported:

*“We take one or two pieces of new fruit for kids to try.” WL2 (Low Fiber)*

Low-fiber participants also highlighted the psychological burden associated with continuous caregiving responsibilities, where household obligations often took precedence over personal health needs. One participant reflected:

*“Even if I'm not well, I have to manage.” WL5 (Low Fiber)*

These findings indicate that family support, caregiving demands, parental food practices, and emotional responsibilities collectively influence dietary behaviors, either facilitating or constraining the adoption of healthier eating patterns.

#### Family-influenced dietary decisions.

Family preferences, particularly those of children, emerged as an important influence on household food choices and meal preparation practices across both fiber intake groups. Participants reported adapting meals to accommodate individual preferences, highlighting the central role of caregiving responsibilities in dietary decision-making.

Among high-fiber participants, food preparation was often tailored to children's requests while remaining integrated within routine home-cooked meals. One participant stated:

*“If my son asks for cucumber dosa with jaggery, I prepare it for him.” WH3 (High Fiber)*

Similarly, low-fiber participants described modifying meals and preparing separate food portions to meet family members’ preferences. As one participant explained:

*“If my son wants some food, I take it for him... I prepare separate portions based on preferences.” WL6 (Low Fiber)*

These findings suggest that children’s food preferences substantially influenced meal planning and preparation across households, shaping everyday dietary practices regardless of fiber intake status.

**Fig 2:**
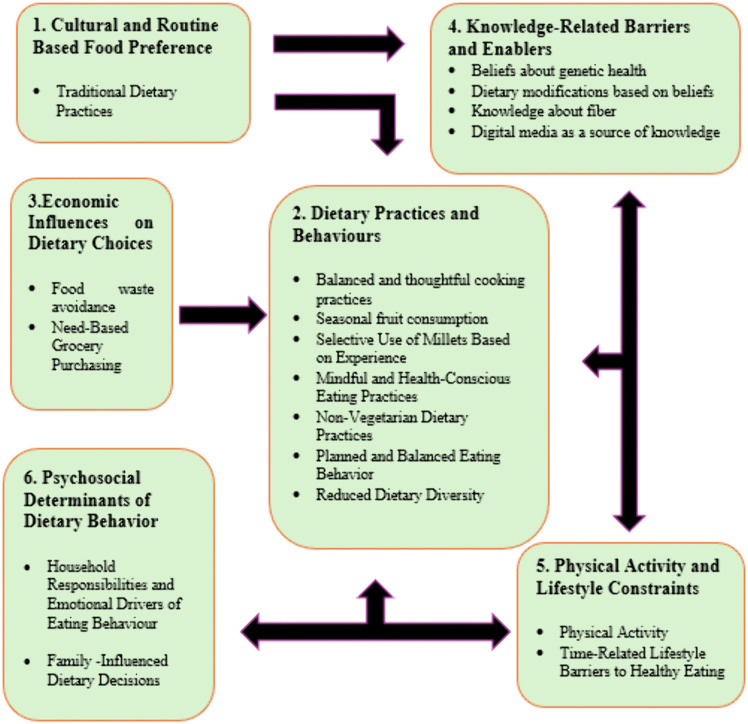
Conceptual framework illustrating factors influencing dietary fiber consumption among participants.

To better illustrate the relationships between the themes identified in this study, a conceptual thematic framework was developed based on the thematic analysis. Fig 2 presents the conceptual framework derived from the thematic analysis. The figure illustrates the key factors influencing dietary fiber consumption among middle-aged women, including cultural/routine-based preferences, dietary practices/behaviours, economic influences, knowledge-related factors, physical activity/lifestyle constraints, and psychosocial determinants. These factors interact to shape participants’ dietary behaviors and influence their ability to adopt higher dietary fiber intake.

Conceptual framework illustrating the factors influencing dietary fiber consumption among participants is presented in Fig 2. The figure illustrates the relationships among the key themes identified in the thematic analysis. Dietary fiber intake behaviors were shaped by multiple interacting determinants, including cultural and routine-based food preferences, dietary practices and behaviors, economic influences, knowledge-related factors, physical activity and lifestyle constraints, and psychosocial determinants. Knowledge and awareness about fiber-rich foods motivated healthier dietary choices among participants; however, these choices were also influenced by household food decision-making, cultural food traditions, affordability of foods, and practical barriers such as time constraints and family preferences. Together, these interconnected factors influenced participants’ ability to incorporate fiber-rich foods into their daily diets.

Fig 2 Conceptual framework of factors influencing dietary fiber intake.

## Discussion

The present study demonstrates that dietary fiber intake among middle-aged South Indian women is influenced by a complex interplay of individual, household, sociocultural, and environmental factors rather than nutritional knowledge alone. Consistent with socio-ecological models of health behavior, participants’ dietary practices were shaped by the interaction between personal beliefs, family dynamics, cultural traditions, economic realities, and lifestyle demands. These findings support growing evidence that dietary behaviors are embedded within broader social and environmental contexts and cannot be fully explained through individual-level determinants alone.

Women reporting higher fiber intake appeared to benefit from household environments that facilitated healthier eating practices, including greater autonomy in food preparation, structured meal routines, and supportive family practices. Similar associations between women’s decision-making power, household food control, and dietary quality have been reported in previous studies examining women’s empowerment and dietary diversity [17]. The findings suggest that the translation of nutritional knowledge into actual dietary behavior depends not only on awareness but also on the availability of social and practical support within the household.

Conversely, lower fiber intake was frequently associated with competing domestic and occupational responsibilities, irregular meal schedules, and time constraints. These observations are consistent with earlier research showing that women often experience difficulties prioritizing their own nutritional needs while fulfilling caregiving and household obligations [5]. Such findings highlight the persistent gendered nature of food-related labor, where responsibility for family meals may coexist with barriers to maintaining personal dietary health. The results therefore reinforce evidence that nutritional vulnerability among women is closely linked to broader social and caregiving expectations rather than solely to individual food choices.

The study also reflects ongoing dietary transitions occurring across India and other rapidly urbanizing settings. Participants who maintained traditional dietary practices, including regular consumption of millets, legumes, seasonal foods, and vegetarian meal patterns, generally demonstrated more favorable fiber intake behaviors. In contrast, increased reliance on convenience-oriented food choices and changing lifestyle patterns appeared to reduce adherence to traditional dietary practices among some participants. These findings align with previous literature describing the nutrition transition in low- and middle-income countries, where traditional fiber-rich dietary patterns are increasingly displaced by refined, energy-dense, and ultra-processed foods [4,18]. Similar trends have been documented in Indian populations experiencing rapid urbanization and changing food environments, resulting in reduced consumption of whole grains, fruits, vegetables, and other fiber-rich foods.

An important contribution of the present study is the demonstration that women’s dietary decisions are often socially negotiated within family systems. Meal preparation and food selection were frequently influenced by the preferences of spouses, children, and other household members, requiring women to adapt their own dietary choices accordingly. This observation supports previous qualitative research highlighting the role of sociocultural norms, family relationships, and household food practices in shaping eating behaviors [12].It further extends existing evidence by illustrating how these influences operate in everyday household contexts and may directly affect women’s ability to maintain fiber-rich dietary patterns. The findings suggest that interventions targeting dietary improvement may achieve greater effectiveness when family members are engaged alongside women rather than focusing exclusively on individual behavior change.

Economic considerations emerged as another important influence on dietary behavior. Although participants generally recognized the value of healthy eating, food purchasing decisions were frequently guided by affordability, accessibility, storage convenience, and preparation time. These findings are consistent with previous studies indicating that financial and practical constraints often influence food choices even among individuals who possess adequate nutritional knowledge. Importantly, the present findings suggest that economic barriers extend beyond income alone and encompass the broader management of household labor, caregiving responsibilities, and time scarcity. Such observations reinforce the need to conceptualize food choice as a multidimensional process influenced by both material and social resources.

Differences in perceptions of hereditary risk and personal responsibility for health further highlight the importance of psychosocial factors in dietary behavior. Participants who viewed family history of obesity or chronic disease as a modifiable risk factor appeared more motivated to adopt preventive dietary practices, whereas others perceived hereditary influences as largely unavoidable. This finding is consistent with behavioral theories suggesting that perceived control and self-efficacy influence engagement in health-promoting behaviors. Greater perceived agency may therefore facilitate healthier dietary choices even in the presence of environmental constraints.

Collectively, these findings contribute to the growing body of literature examining dietary behavior among women in transitional food environments. By providing qualitative insights into the lived experiences of middle-aged South Indian women, the study demonstrates how cultural traditions, household structures, economic realities, caregiving responsibilities, and personal beliefs interact to influence fiber consumption. The findings reinforce evidence that dietary behaviors are shaped by multiple interconnected determinants operating across individual, interpersonal, and environmental levels.

From a practical perspective, interventions aimed at improving fiber intake should move beyond nutrition education alone and adopt family-centered, gender-responsive, and culturally contextualized approaches. Educational strategies should address common misconceptions regarding fiber sources while promoting locally available fiber-rich foods such as millets, legumes, fruits, vegetables, and traditional whole-grain preparations [19] Community-based programs may benefit from incorporating practical meal-planning support, time-saving food preparation techniques, and family-oriented nutrition counseling that encourages shared responsibility for food-related tasks. Strengthening food literacy among all household members may help create supportive environments that facilitate healthier dietary practices.

At the policy level, multisectoral approaches integrating nutrition education with agricultural and food-system initiatives are warranted. Efforts to improve the accessibility, affordability, and acceptability of traditional fiber-rich foods may help counter the dietary shifts associated with urbanization and modernization. Policies promoting women’s empowerment, equitable household food responsibilities, and supportive community networks may further contribute to sustainable improvements in dietary quality. Recognizing the collective nature of food decision-making within households is likely to be particularly important for designing interventions that are both culturally appropriate and effective in promoting long-term dietary behavior change.

### Strengths

This study's primary strength lies in its exploratory qualitative design, providing rich, in-depth insights into women's lived experiences and perceptions regarding fiber consumption. Using native language (Kannada) for interviews fostered participant comfort and facilitated candid expression. The comparison between high and low fiber intake groups allowed for identifying clear differentiators in behaviors and beliefs. Adherence to COREQ guidelines enhances the transparency and rigor of the reporting.

#### Limitations.

This study has several limitations that should be considered when interpreting the findings. The relatively small sample size (n = 13) limits the transferability of the findings to broader populations of South Indian women. Participants were recruited from a hospital-based setting, which may introduce selection bias, as these women may have greater health awareness or existing health concerns compared with the general population. Dietary fiber intake was assessed using a single 24-hour dietary recall, which may be subject to recall bias and may not fully capture habitual dietary intake. Although participants were categorized into high-fiber and low-fiber groups based on reported intake, the qualitative design focused on exploring perceptions and experiences rather than making quantitative comparisons between groups. As with most qualitative studies, the findings are context-specific and are intended to provide in-depth insights into perceptions, barriers, and facilitators of dietary fiber intake rather than statistically generalizable results. Despite these limitations, the study provides valuable contextual understanding of the sociocultural, economic, and behavioral factors influencing dietary fiber consumption among middle-aged South Indian women.

#### Scope for future research.

Future research could involve larger-scale qualitative studies across different demographics and regions of India to assess the transferability of these findings. Quantitative studies could explore the prevalence of identified barriers and enablers. Intervention studies designed based on these qualitative insights, testing the effectiveness of culturally and contextually relevant strategies to increase fiber intake, would be valuable. Further investigation into the psychosocial stressors and their direct impact on dietary choices is also warranted.

## Conclusions

This exploratory qualitative study provides contextual insight into the factors influencing dietary fiber intake among a small group of middle-aged South Indian women recruited from a tertiary care hospital setting. The findings highlight the complex interaction of cultural practices, household dynamics, lifestyle constraints, economic considerations, and health-related beliefs in shaping dietary behaviors. Given the qualitative design and the relatively small, hospital-based sample, the findings should be interpreted within the specific sociocultural and clinical context of the study and are intended to provide conceptual and contextual understanding rather than population-level generalization to the broader South Indian female population.

## Supporting information

S1 Text Semi-structured interview guide used for qualitative interviews. (DOCX)

S1 Table Codebook used for thematic analysis. (ODT)

S2 Table COREQ checklist. (DOCX)

S3 Table SPIRIT checklist. (DOCX)

S4 Table Consort checklist. (DOCX)

S2 Text Study protocol and institutional ethics committee approval. (ODT)

## Abbreviations

BMI: Body Mass Index
GPAQ: Physical Activity Questionnaire
HOMA-IR: Homeostatic Model Assessment of Insulin Resistance
NCDs: Noncommunicable Diseases
NVivo: Qualitative Data Analysis Software
SD: Standard Deviation
WHO: World Health Organization
