# Supplementary material for: Perceptions and barriers to dietary fiber intake among middle-aged south Indian women: A qualitative study
Source: PLOS Glob Public Health. 2026 Jun 24;6(6):e0006685. doi: 10.1371/journal.pgph.0006685 (PMC13293455; doi:10.1371/journal.pgph.0006685)
Supplement: S1 Text — (DOCX) [file pgph.0006685.s001.docx]

| **QUESTIONS**  **IN-DEPTH INTERVIEW QUESTIONNAIRE** | | **PROBE**  Date:  Start Time:  End Time:  Date :  Start Time:  End Time:  **IN-DEPTH INTERVIEW QUESTIONNAIRE**  **INTERVIEW QUESTIONNAIRE** |
| --- | --- | --- |
| **Thank you for agreeing to participate in this interview. How are you doing today?** | | |
| **Demographic factors**   1. **Can you tell me a little bit about yourself?** | Age, Place of Residence, Height, Weight Education, Occupation, Family income, Marital status | |
| **Knowledge-related barrier**   1. What do you know about a healthy diet? | What kinds of foods should we include in our diet for optimal health? | |
| 1. Can you list some common food sources that contain fiber? | Are there any specific health benefits of fiber that you're aware of? | |
| **Dietary practices**   1. Can you describe your dietary habits and typical daily food intake? | How many meals/snacks do you eat per day?  What do you usually have for breakfast, lunch, dinner, and snack options?  What are the most common cooking methods you use at home (eg: baking, frying, steaming)? Why do you prefer these?  How often do you eat out? When eating out, what are the main factors influencing your choice of meal ( taste, convenience, nutrition)  Do you have the habit of eating leftover foods? | |
| 1. What are the cereals, pulses, milk products, and miscellaneous that are commonly consumed in your household? | How often do you prefer these? Do you have any favorite ways of cooking them?  Are there specific ingredients you prioritize for their nutritional benefits? If so, which ones and why? | |
| 1. How often do you consume fruits and vegetables? | Can you describe a typical day's intake of fruits and vegetables? | |
| **Time-related barriers**   1. How does your daily schedule affect your ability to eat healthy meals? | How do commitments and responsibility influence your dietary habits, particularly regarding fiber consumption?  Are there certain days or times when cooking becomes especially difficult?  When you’re busy, how do you usually approach deciding which meals to focus on most?  What strategies do you use to manage your time and prioritize healthy eating? | |
| **Cultural-related barriers :**   1. How do cultural factors influence your food choices and eating habits? | Can you provide a specific example of a cultural tradition or belief that influences your eating habits?  Are there any foods that are a family favorite or part of a traditional recipe?  Have these beliefs or practices been passed down through your family? Do you follow them similarly or have you adapted them?  Have you modified any traditional recipes to meet health goals or dietary needs? | |
| 1. How do social/family pressures or expectations influence your food choices? | Are there any specific foods that you feel pressured to eat or avoid due to social or family influences? | |
| **Psychological Barriers:**   1. How does stress or fatigue impact your food choices during the day? | Are there specific foods you reach for when you’re feeling stressed?  How do you feel if your eating routine is disrupted? Does it affect your mood or energy levels?  Is it important to you to have a specific eating routine, like eating at the same times each day?  How often do you compromise on your meal choices for your family/kids' preferences? | |
| **Economic Barriers:**   1. What criteria do you use when purchasing groceries? 2. How important are financial reasons as a barrier to consuming enough fiber as a healthy food practice? | How does your budget impact the types of food you buy?  When shopping on a budget, do you prioritize quantity over quality or vice versa?  How does budgeting affect the types of food you buy for your family members?  Are there specific nutrition goals you feel are harder to achieve due to cost?  How do you balance the cost of healthy foods compared to other expenses/unhealthy options? | |
| Is there anything else that you want to add to the discussion that we talked about, especially regarding healthy food/ fiber consumption. | | |
| Thank you for your time and participation in this interview. Your insights and experiences have been invaluable. | | |
