# Supplementary material for: Perceptions and barriers to dietary fiber intake among middle-aged south Indian women: A qualitative study
Source: PLOS Glob Public Health. 2026 Jun 24;6(6):e0006685. doi: 10.1371/journal.pgph.0006685 (PMC13293455; doi:10.1371/journal.pgph.0006685)
Supplement: Supplementary file 6 [file pgph.0006685.s002.odt]

| CODEBOOK Theme | Subtheme | Definition | Example (Verbatim Quote) | Group (High/Low Fiber) | Keywords |
| --- | --- | --- | --- | --- | --- |
| Cultural and Routine-Based Food Preferences Traditional Dietary Practices | Traditional Dietary Practices | This subtheme captures the influence of inherited food traditions such as vegetarianism, fasting, festival food restrictions, and health-based recipes that align with traditional cultural modifications. | High fiber intake was associated with consistent cultural adherence, whereas low fiber intake showed attrition of these practices due to convenience and changing family dynamics. We mostly eat vegetarian food and rarely consume non-vegetarian dishes. We have non-veg only on Sundays.” – WH1 (High Fiber) “Only on Fridays my mother-in-law follows vegetarian food...” – WL2 (Low Fiber) “During festivals like Ganesh Chaturthi, we don’t use onion and garlic.” – WH5 (High Fiber) “All of us have already adjusted to today’s lifestyle. There’s nothing like strictly traditional food anymore.” – WL4 (Low Fiber) “We mainly consume ragi... even kids drink ragi before school.” – WH1 (High Fiber) | High and Low Fiber | Fasting, Vegetarian Meals, Cultural Food Rules, Festival Restrictions, Ragi, Jaggery Substitution, Tradition, Recipe Modification, Modern Lifestyle Adaptation |
| Dietary Practices and Behaviours | Balanced and Thoughtful Cooking Practices | Refers to the choice of cooking techniques and preparation habits that reflect nutritional awareness and health orientation. | High fiber participants deliberately used methods like boiling and steaming to retain nutrients and reduce fat, whereas low fiber participants typically selected cooking methods based on ease and availability, often without considering nutritional value or fiber preservation. We mostly use boiling as our primary cooking method… occasionally steam food… like pundi.” – WH1 (High Fiber) “Whatever is easily available, like fruits or vegetables, we just get that and cook.” – WL2 (Low Fiber) | High Fiber and Low Fiber | Boiling, Steaming, Cooking Method, Nutrition-Conscious, Healthy Preparation, Convenience, Ease, Unplanned Cooking, Dietary Awareness |
|  | Seasonal Fruit Consumption | This subtheme refers to the use of seasonal fruits in the daily diet. | High fiber participants showed more consistent integration of seasonal fruits, reflecting a routine habit that supports fiber intake and dietary diversity. Low fiber participants also consumed fruits, but did so with less regularity and intention. Sweet lime and oranges are on daily basis...” – WH7 (High Fiber) | High Fiber | Seasonal Fruits, Citrus, Dietary Routine, Fruit Intake, Food Variety, Fiber Sources, Health Awareness |
|  | Selective Use of Millets Based on Experience | This subtheme reflects participants' millet consumption choices, which were often guided by personal experience or health awareness. | High fiber participants included millets such as ragi intentionally for their health benefits, while low fiber participants showed minimal or inconsistent use, often due to children's taste preferences or lack of familiarity. The data suggest that while millets are recognized as healthy, practical barriers limit their regular inclusion in some households. My sister gave me a millet powder for immunity... I mix 1.5 spoons with milk every day.” – WH7 (High Fiber) “I make things using wheat and jowar; ragi is used less... kids don’t like it.” – WL4 (Low Fiber | High Fiber and Low Fiber | Millets, Ragi, Jowar, Health Benefits, Taste Preferences, Food Familiarity, Traditional Grains, Immunity, Selective Consumption |
|  | Mindful and Health-Conscious Eating Practices | This subtheme captures intentional dietary behaviours aimed at promoting health. | High fiber participants engaged in mindful practices such as consuming herbal drinks (e.g., jeera Kashaya) and incorporating functional foods into daily routines. In contrast, low fiber participants reported food or beverage consumption (e.g., tea) primarily as a coping mechanism for stress, rather than with nutritional goals. “After washing his hands, my husband drinks jeera kashaya.” – WH1 (High Fiber) “I drink tea mainly to relax when I’m stressed.” – WL6 (Low Fiber) | High Fiber and Low Fiber | Mindful Eating, Herbal Drinks, Jeera Kashaya, Intentional Choices, Stress Relief, Tea, Health-Oriented, Functional Foods, Nutrition Consciousness |
|  | Non-Vegetarian Dietary Practices | This subtheme explores how non-vegetarian food is incorporated into daily diets and how it affects fiber intake. | High fiber participants demonstrated moderation and balance, often including vegetables alongside non-veg items and focusing on portion control. In contrast, low fiber participants consumed non-vegetarian food more frequently and without vegetable accompaniments, reducing overall fiber consumption. “We eat non-veg 5–6 days a week.” – WL2 (Low Fiber) “It is better to include more leafy greens and vegetables in the diet... I believe that eating eggs in moderation is fine.” – WH2 (High Fiber) | High Fiber and Low Fiber | Non-veg, Meat, Eggs, Vegetables, Fiber reduction, Portion control, Balance, Moderation, Meal composition, Dietary pattern |
|  | Planned and Balanced Eating Behaviour | This subtheme captures the role of meal planning and structured eating routines in dietary quality. | High fiber participants demonstrated proactive strategies like soaking ingredients in advance, which helped maintain regular and balanced meals. In contrast, low fiber participants frequently skipped meals or relied on less nutritious alternatives due to time constraints and a lack of planning. “I soak rice and urad dal together… keep it overnight.” – WH6 (High Fiber) “I eat breakfast only when I have time; otherwise, I go for a snack or just stick to three main meals.” – WL4 (Low Fiber) | High Fiber and Low Fiber | Meal preparation, Planning, food routine, Time management, skipped meals, Unplanned eating, Fiber routine, Structured diet, Dietary regularity |
|  | Reduced Dietary Diversity | This subtheme addresses the lack of variety in daily meals. | Their diets were often repetitive and based on simple staples, lacking the inclusion of diverse fiber-rich foods. This monotony is closely linked to lower fiber intake and represents a potential area of nutritional deficiency and vulnerability. “We usually don’t make too many varieties, mostly we prepare simple meals like rice ganji. Once we make ganji, we eat for a couple of days.” – WL3 (Low Fiber) | Low Fiber | Monotony, Simple Meals, Rice Ganji, Repetitive Eating, Low Variety, Limited Fiber, Meal Repetition, Poor Dietary Diversity |
| Economic Influences on Dietary Choices | Food Waste Avoidance | This subtheme highlights strategies used to reduce food waste, reflecting economic decision-making. | High fiber participants used structured planning to purchase only necessary fresh items, supporting fiber intake. Low fiber participants relied on homegrown or loose local produce without strict quality checks, which may limit dietary diversity. “We buy only what we need for the day so that nothing goes to waste and we eat fresh vegetables daily.” – WH3 (High Fiber) “We grow vegetables at home… we avoid waste and eat them.” – WL4 (Low Fiber) “We don’t go to check the quality; everything is brought loose like half kg at a time.” – WH3 (Low Fiber) | High Fiber and Low Fiber | Food Waste, Planning, Homegrown, Local Produce, Fresh Vegetables, Unstructured Buying, Quality, Economic Choice |
|  | Groceries & Need-Based Purchasing | This subtheme explores how participants purchase groceries based on economic considerations and storage constraints. | High fiber participants focused on both cost-effectiveness and nutritional value, favouring fiber-rich, high-quality items. In contrast, low fiber participants made small, practical purchases, emphasizing ease of storage and immediate usability rather than planned nutrition. “Whatever is cost-effective and nutritious, we purchase more of it.” – WH1 (High Fiber) “We make sure the groceries are of good quality and enough for use, but we don’t buy in large quantities because it’s easier to store when we buy only what’s needed.” – WL2 (Low Fiber) | High Fiber and Low Fiber | Nutrition, Affordability, Cost-Effective, Small Quantity, Storage, Quality, Grocery Planning |
| Knowledge-Related Barriers and Enablers | Beliefs About Genetic Health | This subtheme explores participants’ perceptions of heredity as a factor in obesity. . | While both groups recognized genetic predisposition, high fiber participants used this awareness to adopt healthier eating habits. In contrast, low fiber participants saw heredity as inevitable, reducing motivation for dietary change “I feel my obesity might be hereditary since my mother and sibling are also overweight, so I make a conscious effort to eat healthy by including more vegetables, pulses, fruits, and other nutritious foods in my diet.” – WH2 (High Fiber) “Speaking about obesity, I have been gaining weight recently. Previously as well, and my father is obese, so it could be hereditary.” – WL6 (Low Fiber) | High Fiber and Low Fiber | Heredity, Obesity, Genetic Risk, Motivation, Awareness, Passive Acceptance, Dietary Behavior |
|  | Dietary Modification Based on Beliefs | This subtheme highlights participants' partial understanding of dietary fiber sources. . | In this group, fiber was commonly associated only with fruits, vegetables, and greens, excluding other key sources like whole grains and pulses. Such limited beliefs may contribute to lower fiber intake “According to me, fiber is in fruits and vegetables… also leafy greens.” – WL3 (Low Fiber) | Low Fiber | Fiber Knowledge, Misconceptions, Fruits, Vegetables, Leafy Greens, Limited Awareness, Dietary Belief |
|  | Digital Media as a Source of Knowledge | This subtheme explores the use of digital platforms, especially social media, as a source of dietary and health-related information. . | High fiber participants used such media to seek tips and stay informed, reflecting health motivation, but also posing risks of misinformation due to unverified sources “With health being important, we often turn to social media for tips and advice. Since staying healthy is important, we try to learn more about it.” – WH1 (High Fiber) | High Fiber | Digital Media, social media, Health Tips, Online Information, Self-Education, Misinformation Risk |
|  | Knowledge About Fiber | This subtheme examines the extent of participants’ understanding of fiber-rich foods. | High fiber group participants demonstrated broader knowledge, including traditional sources like ragi, and acknowledged the fiber's role in satiety and digestion. Low fiber participants had limited awareness, mostly identifying only fruits or pulses, with diets dominated by refined rice and fried foods. “Fiber is now ragi… gives us a full feeling…” – WH5 (High Fiber) “The weekly diet is dominated by refined rice-based meals and frequent fried non-vegetarian dishes with limited inclusion of fiber-rich foods.” – WL5 (Low Fiber) “We should use more pulses and legumes because they have more protein and fiber, beyond that, I don’t know much.” – WL5 (Low Fiber) | High Fiber and Low Fiber | Ragi, Satiety, Digestive Health, Rice-Based Meals, Fried Food, Limited Knowledge, Fiber Diversity |
| Physical Activity and Lifestyle Constraints | Physical Activity | This subtheme explores differences in physical activity behaviours between the groups. | High fiber participants incorporated regular activities like yoga and walking into their daily routines, reflecting a holistic approach to health. Low fiber participants reported barriers such as fatigue and domestic workload, leading to inactivity outside work hours. “I wake up at 4:30 am and do 15 minutes of yoga, then go for a walk.” – WH5 (High Fiber) “There’s no extra physical activity… no time or energy left.” – WL3 (Low Fiber) | High Fiber and Low Fiber | Yoga, Walking, Daily Routine, Sedentary Lifestyle, Fatigue, Domestic Workload, Inactivity, Time Constraint |
|  | Time-Related Lifestyle Barriers to Healthy Eating | This subtheme addresses how participants manage or struggle with time in relation to dietary behaviours. | High fiber participants adopted time-saving strategies like prepping vegetables the night before to ensure consistent, healthy meals. In contrast, low fiber participants reported unpredictable mealtimes and difficulty balancing work and home demands, leading to compromised dietary quality “Since we leave early, I chop vegetables the night before.” – WH5 (High Fiber) “Most time goes in office… then at home with chores. Hard to get time for myself.” – WL4 (Low Fiber) “We just eat when the time comes… sometimes no fixed mealtime.” – WL1 (Low Fiber) | High Fiber and Low Fiber | Time Constraint, Meal Preparation, Work-Life Balance, Fixed Mealtime, Erratic Eating, Dual-Role Stressors |
| Psychosocial Determinants of Dietary Behavior | Household Responsibilities and Emotional Drivers of Eating Behavior | This subtheme captures how emotional strain and domestic workload influence dietary behavior. . | High fiber participants benefited from shared responsibilities, enabling structured meal planning and healthier eating. Low fiber participants reported emotional fatigue, lack of support, and reactive eating, often compromising dietary quality and consistency “My mother-in-law and I divide work, so I get some time to plan and cook properly.” – WH1 (High Fiber) “I must manage everything by myself. It becomes hectic.” – WH1 (High Fiber) “We rarely eat outside. We avoid it because the children might develop a habit.” – WH4 (High Fiber) “Only if kids ask, we eat from outside.” – WL2 (Low Fiber) “We take one or two pieces of new fruits for kids to try.” – WL2 (Low Fiber) “Even if I’m not well, I have to manage.” – WL5 (Low Fiber) | High Fiber and Low Fiber | Emotional Fatigue, Caregiving, Shared Responsibility, Children’s Preferences, Outside Food, Lack of Support, Self-Care Burden, Tentative Healthy Eating |
|  | Family-Influenced Dietary Decisions | This subtheme explores how family, especially children's preferences, influence food choices. | High fiber participants made conscious efforts to balance health with family preferences, whereas the low fiber intake people are more likely to react quickly and adapt without focusing on nutrition quality. “If my son asks for cucumber dosa with jaggery, I prepare it for him.” – WH3 (High Fiber) “If my son wants some food, I take it for him... I prepare separate portions based on preferences.” – WL6 (Low Fiber) | High Fiber and Low Fiber | Family Influence, Children’s Preference, Separate Portions, Reactive Decisions, Health Balance |
