# Supplementary material for: Perceptions and barriers to dietary fiber intake among middle-aged south Indian women: A qualitative study
Source: PLOS Glob Public Health. 2026 Jun 24;6(6):e0006685. doi: 10.1371/journal.pgph.0006685 (PMC13293455; doi:10.1371/journal.pgph.0006685)
Supplement: Supplementary file 7 [file pgph.0006685.s006.odt]

TRIAL PROTOCOL

1. Study Title

Perceptions and Barriers to Dietary Fiber Quality and Intake Among Middle-Aged South Indian Women: A Qualitative Study

2. Trial Registration

Registry name: Clinical Trials Registry – India (CTRI)

Trial registration number: CTRI/2022/01/039074

Date of registration: 03 January 2022

Trial registry URL: http://ctri.nic.in

3. Protocol Version and Date

Protocol version number: Version 1.0

Date of current version: January 2022

4. Investigators and Affiliations

Principal Investigator:

Suvarna Hebbar
Assistant Professor Senior Scale,  Clinical Nutrition and Dietetics Program, Manipal College of Health Professions, Manipal Academy of Higher Education, Manipal 576104, Karnataka, India.
Email: suvarna.hebbar@manipal.edu

Corresponding Author: 

Suvarna Hebbar

Assistant Professor Senior Scale,  Clinical Nutrition and Dietetics Program, Manipal College of Health Professions, Manipal Academy of Higher Education, Manipal 576104, Karnataka, India.
Email: suvarna.hebbar@manipal.edu

Latha Thimmappa
Professor, College of Nursing, All India Institute of Medical Sciences (AIIMS), Bibinagar, Telangana, India.
Email: [latharadhakrishna@gmail.com](mailto:latharadhakrishna@gmail.com)

Co-investigators:

Shashikiran Umakanth- Department of General Medicine,Dr. TMA Pai Hospital, Udupi, Melaka Manipal Medical College, Manipal Academy of Higher Education, Manipal, Karnataka, India.

Amrutha M. S- Clinical Nutrition and Dietetics Program , Manipal College of Health Professions, Manipal Academy of Higher Education, Manipal 576104, Karnataka, India.

Ramya Bhagavath- Department of Clinical Nutrition and Dietetics, Kasturba Hospital, Manipal 576104, Karnataka, India.

5. Background and Rationale

Dietary fiber is a critical component of a healthy diet and plays an essential role in the prevention and management of non-communicable diseases such as type 2 diabetes, cardiovascular disease, obesity, and certain cancers. Despite global and national dietary recommendations advocating a daily fiber intake of 25–30 g for adults, fiber consumption remains consistently below recommended levels among Indian populations, particularly among women. Sociocultural norms, time constraints, economic factors, and misconceptions regarding fiber-rich foods contribute to inadequate intake. Middle-aged women, who often serve as primary household food decision-makers, face unique barriers that influence dietary practices. Understanding perceptions, barriers, and enablers related to fiber intake among this population is essential for designing culturally sensitive public health nutrition interventions. This qualitative study was therefore designed to explore these factors among middle-aged South Indian women, comparing high and low dietary fiber intake groups.

6. Study Objectives

6.1 Primary Objective

To explore perceptions, barriers, and enabling factors influencing dietary fiber quality and intake among middle-aged South Indian women.

6.2 Secondary Objectives

To compare perceptions and dietary practices between women with high dietary fiber intake (≥20 g/day) and low dietary fiber intake (<20 g/day).

To identify sociocultural, dietary, economic, lifestyle, and psychosocial determinants influencing fiber intake.

7. Study Design

This study employed an exploratory qualitative design using in-depth, semi-structured interviews. The study followed the Consolidated Criteria for Reporting Qualitative Research (COREQ) guidelines.

8. Study Setting

The study was conducted in the outpatient departments of Medicine, Gynaecology, and Endocrinology at a tertiary care hospital in South India.

9. Study Population

9.1 Inclusion Criteria

- Women aged 35–55 years

- Attending outpatient services at the study hospital

- Willing to provide informed consent

9.2 Exclusion Criteria

- Women with severe acute illness

- Those unwilling to participate or unable to provide informed consent

10. Sample Size and Sampling Strategy

A purposive sampling strategy was employed. A total of 13 participants were included, guided by the principle of data saturation. Participants were categorized into high-fiber (≥20 g/day; n = 7) and low-fiber (<20 g/day; n = 6) groups based on 24-hour dietary recall.

11. Participant Recruitment

Eligible women attending outpatient clinics were approached and informed about the study. Fifteen women were assessed for eligibility, of whom two declined participations due to time constraints. Thirteen women provided written informed consent and were enrolled.

12. Data Collection Procedures

Data were collected using a semi-structured interview guide developed through literature review and expert validation. Interviews were conducted in Kannada, audio-recorded with participant permission, and lasted approximately 30 minutes. Interviews were conducted in a private setting within the hospital premises.

13. Data Management and Confidentiality

Audio recordings and transcripts were anonymized and stored securely with access restricted to the research team. Identifiable information was removed during transcription to ensure confidentiality.

14. Data Analysis Plan

Transcripts were analyzed thematically using NVivo version 15 software. Coding was conducted iteratively to identify patterns and themes. Data saturation was achieved by the 12th interview, with no new themes emerging thereafter.

15. Ethical Considerations

Ethical approval was obtained from the Institutional Ethics Committee of Kasturba Medical College and Kasturba Hospital (IEC approval number: 519/2021). The study was conducted in accordance with the Declaration of Helsinki. Written informed consent was obtained from all participants prior to data collection.

16. Benefits and Risks

Participants did not receive direct clinical benefits. Risks were minimal and limited to potential emotional discomfort during interviews. Participants were informed of their right to withdraw at any time.

17. Compensation and Reimbursement

No monetary compensation was provided to participants.

18. Dissemination of Results

Findings will be disseminated through peer-reviewed publications and scientific presentations.

19. Protocol Amendments

No amendments were made to the study protocol after initial ethics committee approval.

20. Funding and Support

This research was supported under the Department of Health Research (DHR) Grant-in-Aid Scheme (Proposal ID: ICMR-2022-1512).

21. Conflicts of Interest

The investigators declare no financial or non-financial conflicts of interest.

22. References

1.	He Y, Wang B, Wen L, Wang F, Yu H, Chen D, et al. Effects of dietary fiber on human health. Food Sci Hum Wellness. 2022;11(1):1–10. 

2.	Mayor S. Eating more fibre linked to reduced risk of non-communicable diseases and death, review finds. BMJ [Internet]. 2019;364(January). Available from: http://dx.doi.org/doi:10.1136/bmj.l159

3.	Viswanathan B, Agnihotri A. Double Burden of Malnutrition in India: Decadal Changes among Adult Men and Women. 2020;(August):1–56. Available from: https://ideas.repec.org/p/mad/wpaper/2020-200.html

4.	Ramsteijn AS, Louis P. Dietary fibre optimisation in support of global health. Microb Biotechnol. 2024;17(8):1–9. 

5.	Gupta S, Seth P, Vemireddy V, Pingali P. Women’s empowerment and intra-household diet diversity across the urban continuum: Evidence from India’s DHS. Food Policy [Internet]. 2024;128(July):102680. Available from: https://doi.org/10.1016/j.foodpol.2024.102680

6.	Yogal C, Stunes AK, Shakya S, Karmarcharya B, Koju R, Mosti MP, et al. High prevalence of overweight/obesity and central obesity among women in a rural district of Nepal in 2012–2013: a population-based study. Front Public Heal. 2025;13(February):1–12. 

7.	Singh N, Benjamin S, Lakdawala M, Nawathe V. Original Article Fiber Gap in the Daily Diet of Indian Population : A Narrative Review. 2023;4(4):13–8. 

8.	Codex T, Commission A, Codex T, Commission A, Diet HA. POSITION OF THE INDIAN DIETETIC ASSOCIATION : DIETARY FIBRE AND HEALTH December 2018 POSITION STATEMENT POSITION OF THE INDIAN DIETETIC ASSOCIATION : DIETARY FIBRE AND HEALTH. 2018;(December):1–14. 

9.	Agurs-Collins T, Alvidrez J, ElShourbagy Ferreira S, Evans M, Gibbs K, Kowtha B, et al. Perspective: Nutrition Health Disparities Framework: A Model to Advance Health Equity. Adv Nutr [Internet]. 2024;15(4):100194. Available from: https://doi.org/10.1016/j.advnut.2024.100194

10.	Nitzko S. Consumer preferences for naturally high-fiber and fiber-fortified foods: a preliminary segmentation study. J fur Verbraucherschutz und Leb [Internet]. 2023;18(2):119–32. Available from: https://doi.org/10.1007/s00003-023-01429-w

11.	Tong A, Sainsbury P, Craig J. Consolidated criteria for reporting qualitative research (COREQ): A 32-item checklist for interviews and focus groups. Int J Qual Heal Care. 2007;19(6):349–57.
